# The use of information and communication technologies in Latin American dentists: a cross-sectional study from Ecuador

**DOI:** 10.1186/s12903-020-01137-z

**Published:** 2020-05-19

**Authors:** Ivan Chérrez-Ojeda, Carlos Vera, Emanuel Vanegas, Juan Carlos Gallardo, Miguel Felix, Fernando Espinoza-Fuentes, Peter Chedraui, Antonio W. D. Gavilanes, Valeria L. Mata

**Affiliations:** 1 Universidad Espíritu Santo, Km. 2.5 vía La Puntilla, Zip code: 0901-952 Samborondón, Ecuador; 2 Respiralab Research Group, Guayaquil, Ecuador; 3 Facultad De Ciencias Médicas, Instituto De Investigación e Innovación En Salud Integral, Universidad Católica De Santiago De Guayaquil, Guayaquil, Ecuador; 4 Facultad De Ciencias De La Salud, Universidad Católica “Nuestra Señora De La Asunción”, Asunción, Paraguay; 5 School of Oncology and Developmental Biology, Maastricht University, Maastricht, the Netherlands; 6 Department of Pediatrics, Maastricht University Medical Center, Maastricht, the Netherlands

**Keywords:** Dentists, Ecuador, Information and communication technologies, Latin America, Social media

## Abstract

**Background:**

The use of information and communication technologies (ICTs) provide the tools for enabling fast and reliable real-time communications, as well as the transfer of information between dental professionals and their patients. However, little is known about the frequency and preference of ICTs among Latin-American dentists. Our study aims to fill this gap by assessing different aspects related to ICTs, mainly the frequency of use, perceptions, and barriers among Ecuadorian dentists.

**Methods:**

An anonymous, cross-sectional survey-based study was conducted among 342 Ecuadorian dentists. The final questionnaire included 13 items related to the frequency of use, perceptions, and barriers of ICTs. Bivariate analysis was performed by using chi-squared testing to explore the association between the independent variables and the intended use of ICTs, as well as to characterize the perceptions and barriers related to ICTs.

**Results:**

In general, most participants reported the use of ICTs to communicate with colleagues (99.7%), and patients (96.2%), while only 63.5% reported using ICTs to obtain academic information in their daily practice. WhatsApp was rated as the most used ICT for communicating with colleagues and patients. A majority of participants considered that ICTs can be useful for facilitating continuing dental education (92.1%), searching new work opportunities (91.5%), promoting health (90.1%), working with colleagues and other health professionals (91.2%), promoting their professional services (90.6%), and for resolving clinical cases (87.7%). On the subject of barriers, privacy and security concerns about personal and/or patient information was the biggest concern among dentists (65%), followed by lack of time to learn how to use and/or use ICTs (48%), lack of mobile internet access (28.1%), and lack of internet access at work (24.9%).

**Conclusion:**

In our study, we found that Ecuadorian dentists had a high usage rate of ICTs, mainly for communicating with other colleagues and patients, while the academic use of technology remains a comparatively underused application. Most of the participants surveyed had a positive perception towards ICTs, while privacy and security concerns were identified as the main barrier. Older age was associated with a less favourable perception toward ICTs, as well as an increased likelihood of reporting barriers related to the use of technology.

## Background

Information and communication technologies (ICTs) in a broad sense are defined as the digital technologies supporting the capture, processing, storage and exchange of information [1]. The popularity of these tools has created a need for healthcare providers to have an online presence and interact with their patients via an increasing number of channels, constituting a new domain called e-health [2]. Among the benefits of ICTs are the opportunity to improve efficiency, reduce costs, and expand patient coverage [3]. Furthermore, studies have found that patients are using social media as a tool to connect with other patients and create a support network of individuals living with similar conditions [4]. This has created a new paradigm in which patient interactions extend beyond the confines of the office visit [5].

In emerging societies digital technologies, particularly, smartphones have been adopted extensively [6]. For instance, in 2018 41.4% of the Ecuadorian population owned a smartphone, up from 6.2% in 2012, while mobile devices accounted for approximately 24% of web traffic in the country [7]. This has allowed communities to leapfrog barriers related to the installation of infrastructure, allowing them to participate in the digital environment. On this regard, the World Health Organization (WHO) considers that developing the infrastructure of ICTs for the healthcare system is essential for promoting equitable, affordable, and universal access [8]. Furthermore, the American Dental Education Association (ADEA) considers communication skills as a core area in behavioural sciences in the dental curriculum, and recently defined standards for teaching and assessing competencies in communication skills for patient education and health promotion [9].

The increased use of these tools, and specifically the wealth of information available online, has changed the behaviour of many patients. This new patient group called the *e-patients*, also referred to as *internet patients*, is actively engaged in their health care and health decision making processes [10]. They value above all else autonomy, choice, and vigorously perform their health information-gathering online [10]. There is evidence showing that *e-patients* have refused or terminated recommended dental treatments because of the information they found online [11]. Therefore, the role of dentists in health communication is changing, and they need to leverage ICTs to improve communication with their partners and patients.

Despite the theoretical benefits that ICTs can bring into the practice of dentistry, it is necessary to be aware of the responsibilities these technologies entail, in particular the protection of patient information, and the need for the establishment of a professional standard for interacting through such channels [12]. Based on this, our study aims to provide a better understanding on how Ecuadorian dentists relate to different aspects of ICTs in their daily practice, such as the frequency of use, perceptions and barriers. Our goal is to provide data that generates insights to facilitate the implementation of ICTs into real-life dentistry practices.

## Methods

### Study design and population

An anonymous, cross-sectional survey-based study was completed by 342 Ecuadorian dentists. We adapted the questionnaire from a previous survey used in physicians [13]. The questionnaire surveyed demographic characteristics and recorded the frequency of use, perceptions and barriers of ICTs using a 5-point Likert scale. Frequency of use responses for each ICT ranged from “Never” to “Daily Use”. Perceptions and barriers for ICTs were recorded using a 5-point Likert scale ranging from “Strongly Disagree” to “Strongly Agree”. The ICTs recorded were classified according to their intended use: “Communicating with Patients”, “Interacting with patients”, “Communicating with Colleagues”, “Interacting with Colleagues” and “Academic Information Searching”.

The ICTs included in the questionnaire for *“Communicating with Patients” and “Communicating with Colleagues”* were SMS, email, Line, WhatsApp, Hangouts, Vibe, Facebook messenger, and Telegram. For “*Interacting with patients” and “Interacting with Colleagues”* Blogger, Facebook, Google Plus, Instagram, Snapchat, Tumblr, Twitter, and YouTube were included. Finally, for *“Academic Information Searching”* we included Academia.edu, Google Scholar, Medscape, PubMed, ResearchGate, Scopus, UpToDate, and Cochrane.

### Sample size calculation

We estimated the sample size based on the statistical test with the biggest sample requirement, which in this case were the bivariate logistic regressions. Based on a previous work by Bujang and colleagues, the sample size was calculated using the following formula [14]:

$$ n=100+50i $$

Where *i* refers to the number of independent variables in the final model. Applying this method, we found that a bivariate logistic regression with 4 independent variables would need a minimum sample of 300 in order to represent the parameters.

### Ethical considerations

The study was approved by the ethics committee ‘Comité de ética e Investigación en Seres Humanos (CEISH)’. The survey was anonymous, and the identity of dentists who participated in the study was not revealed and personal data protection was conserved. Written informed consent for the use of the information recorded was obtained before participating in the survey.

### Statistical analysis

Demographic data was explored by calculating frequencies and percentages for categorical variables and mean and standard deviation (SD) for continuous variables. According to the distribution, age was dichotomized as less than 33 years old and equal to or more than 33 years old. Similarly, years of practice were dichotomized as less than 8 years and equal or more than 8 years.

Items pertaining to perceptions and barriers upon the use of ICTs were dichotomized into “disagree” (strongly disagree and disagree) or “agree” (neutral, agree and strongly agree). ICTs were grouped according to their intended use. For each ICT, frequency of use was divided between “No Use”, “Low Frequency”, and “High Frequency”. If a respondent recorded “High Frequency” on any ICT within a use group, they were classified as a High Frequency user for that purpose. If no instances of “High Frequency” were found, responses were checked for instances of “Low Frequency” and classified accordingly. Otherwise, the respondent was classified as “Non-User” for that group of ICTs.

In order to perform the multivariate analysis, the frequency of use of ICTs for each intended purpose were dichotomized between User and Non-User for “*Communicating with Patients”, “Communicating with Colleagues”, and “Interacting with Colleagues”.* On the other hand, “*Interacting with patients” and “Academic Information Searching”* were was dichotomized between “*High Frequency” and “No use/Low Frequency”* because there were an insufficient amount of cases of “*Non Users*” in these groups.

Bivariate analysis was performed between independent variables by using chi-squared testing to explore the association between the independent variables (age, gender, postgraduate years, location, type of institution, specialist degree), and the intended use of ICTs, as well as to characterize perceptions and barriers of ICT use. Variables with a significant *p* value were selected for regression analysis. Generalized linear models were created for each ICT use, perception and barrier. Odd ratios and 95% confidence intervals were computed. A p value < 0.05 was considered significant for the final analysis. Analysis was conducted in R (R Core Team, 2014 [15].

## Results

342 participants were included in the final analysis. The mean age was 37 years, with 12.4 years of practice as average. Female dentists represented 51.8% of the sample. Most participants were general dentists (61.1%), worked in private service (67%) and in an urban setting (93%). Regarding access to smartphones, 97.66% of dentists reported owning one.

### Frequency of use of information and communication technologies among Ecuadorian dentists

**Fig. 1:**
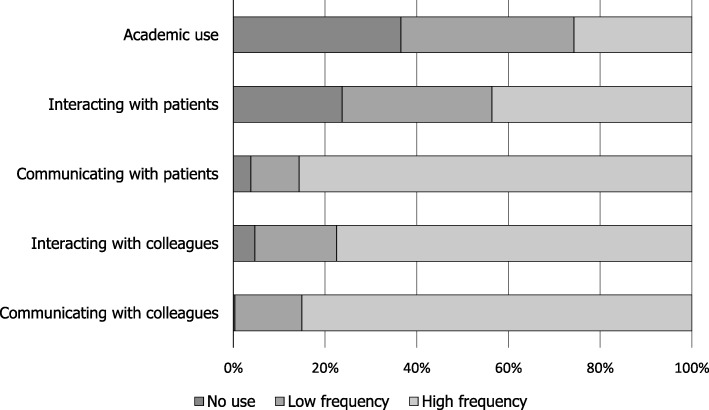
Frequency of use of information and communication technologies for academic purposes, and patient/colleague communication and interaction among Ecuadorian dentists

In general, most participants reported the use of ICTs to communicate with colleagues (99.7%), and patients (96.2%), while only 63.5% reported using ICTs to obtain academic information in the daily practice (Fig. 1). Furthermore, most dentists reported a high use of ICTs for interacting with patients (76.3%) and colleagues (95.3%). Discussing specific ICTs, we found that WhatsApp was the most used ICT, with 97.4% of participants using it to communicate with colleagues, and 93.6% with patients. Similarly, Facebook was used by 88.0% (*n* = 301) of dentists to interact with colleagues, while 67.8% did so with patients. The frequencies of use of all ICTs studied are depicted in Supplemental Appendix Table S1. ICTs for academic purposes had lower usage rates compared to other categories. In this regard, the most frequently used platform was PubMed with 42.7% (*n* = 146) of respondents, while UpToDate was only used with by 4.4% of surveyed participants (Supplemental Appendix Table S1).

Regarding the use of ICTs, specialists were more likely (OR 4.60) to use them for academic purposes. On the other hand, having more than 8 years of experience was associated with a lower likelihood of using ICTs for academic purposes (OR 0.27), as well as for patient interaction (OR 0.38). Variables associated with an increased likelihood of using ICTs for communicating with patients were female gender (OR 2.81), specialists (OR 3.14), and working at private institutions (OR 2.17) (Table 1).

**Table 1:** Generalized logistic regression analysis between the use of ICTs and demographic variables for communication, interaction, and academic purposes among Ecuadorian dentists

	Variable	Odds Ratio	95% CI	*p*-value
Communication with patients	Female gender	**2.81**	[1.46–5.65]	< 0.001
	Private institution	**2.17**	[1.14–4.16]	0.02
	Specialists	**3.14**	[1.47–7.32]	< 0.001
Communication with other professionals	Years of Experience > 8	1.14	[0.371–3.63]	0.82
	Age > 33 years	2.15	[0.684–6.85]	0.19
Academic use	Years of Experience > 8	**0.27**	[0.097–0.727]	0.01
	Age > 33 years	0.71	[0.264–1.94]	0.5
	Private institution	1.58	[0.913–2.70]	0.09
	Specialists	**4.6**	[2.59–8.47]	< 0.001
Interaction with patients	Years of Experience > 8	**0.38**	[0.154–0.912]	0.03
	Age > 33 years	0.86	[0.356–2.11]	0.73
	Private institution	**2.45**	[1.50–4.01]	< 0.001
Interaction with other colleagues	Years of Experience > 8	0.97	[0.368–2.53]	0.98
	Age > 33 years	0.97	[0.369–2.55]	0.95
	Private institution	1.04	[0.593–1.79]	0.89

Notes: Reference gender category is male, age category is < 33 years old, and type of practice is general dentist. *ICT* information and communication technology, *CI* confidence interval

### Perceptions and barriers of information and communication technologies among Ecuadorian dentists

**Fig. 2:**
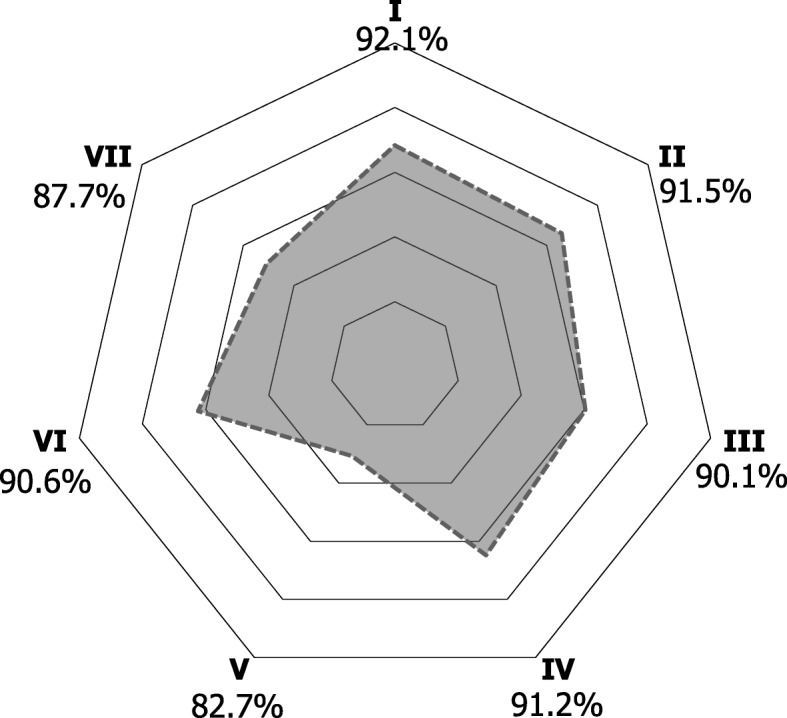
Perceptions of information and communication technologies among Ecuadorian dentists. Each perception is represented by one vertex. All proportions depicted are individuals that agree with the statement. I, “ICTs facilitate continuing dental education” II, “ICTs are useful to search for new work opportunities” III, “ICTs are useful for health promotion” IV, “ICTs are useful for working with colleagues and other health professionals” V, “Prefer ICTs to traditional channels of communication” VI, “ICTs are useful for promoting my professional services” VII, “ICTs can be useful for resolving clinical cases”

**Fig. 3:**
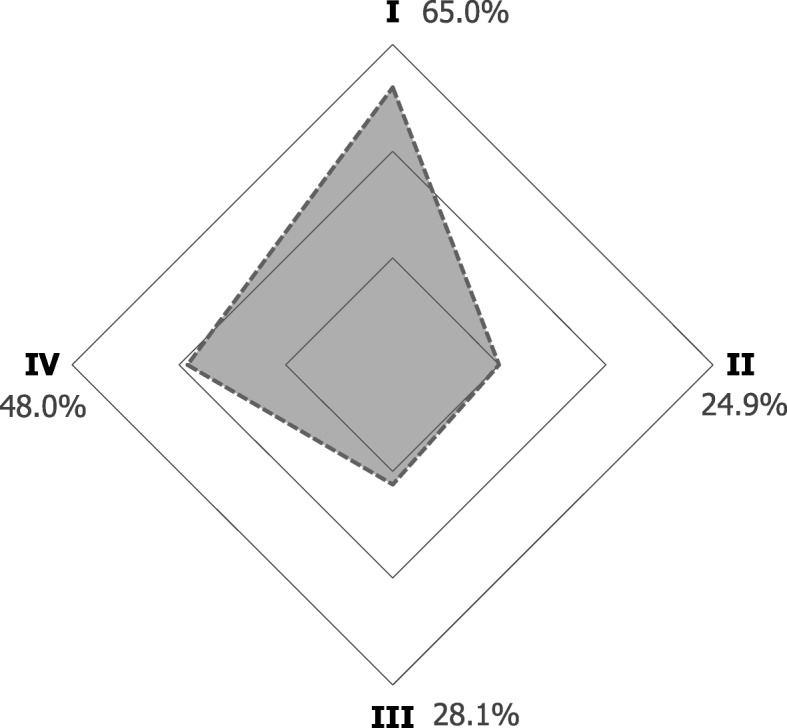
Barriers of information and communication technologies among Ecuadorian dentists. Each barrier is represented by one vertex. All proportions depicted are individuals that agree with the statement. I, ‘Concerned about privacy or security about personal and/or patient information’ II, ‘Do not have access to mobile internet’ III, ‘Do not have access to the internet at work’ IV, ‘Do not have enough time to neither learn how to use them or use them’

In general, most participants agreed that ICTs can be useful for facilitating continuing dental education (92.1%), searching new work opportunities (91.5%), promoting health (90.1%), working with colleagues and other health professionals (91.2%), promoting their professional services (90.6%), and for resolving clinical cases (87.7%); furthermore 82.7% of participants preferred them over traditional channels of communication (Fig. 2). On the subject of barriers, privacy and security concerns about personal and/or patient information was the biggest concern among dentists (65%), followed by lack of time to learn how to use and/or use ICTs (48%), lack of mobile internet access (28.1%), and lack of internet access at work (24.9%) (Fig. 3).

Logistic regression analyses between demographic variables and the perceptions and barriers studied, showed that dentists working at private institutions were more likely to agree that ICTs can be useful for self-promoting services (OR 5.48), solving clinical cases (OR 2.51), and for working with other colleagues (OR 2.25) (Table 2). However, the type of institution had no significant statistical association with any of the barriers presented. In the case of gender, we found that female dentists were more likely to agree that ICTs can be useful for working with other colleagues (OR 3.44), and promoting health (OR 5.74), while also presenting an increased chance of reporting the following barriers: privacy and security concerns (OR 1.89), lack of mobile internet access (OR 1.91), and lack of internet access at work (OR 2.1).

**Table 2:** Generalized logistic regression analysis between specific perceptions of ICTs and demographic variables among Ecuadorian dentists

Perception	Variable	Odds Ratio	95% CI	*p*-value
I. ICTs facilitate continuing dental education	Age > 33 years	**0.16**	[0.039–0.688]	0.01
	Years of Experience > 8	2.37	[0.594–8.41]	0.2
	Female gender	1.55	[0.668–3.81]	0.32
II. ICTs are useful to search for new work opportunities	Age > 33 years	**0.14**	[0.034–0.568]	0.01
	Years of Experience > 8	2.7	[0.679–9.4]	0.13
III. ICTs are useful for health promotion	Age > 33 years	0.55	[0.14–2.13]	0.38
	Years of Experience > 8	0.63	[0.161–2.26]	0.5
	Female gender	**5.74**	[2.32–17.4]	<.001
IV. ICTs are useful for working with colleagues and other health professionals	Female gender	**3.44**	[1.5–8.92]	0.01
	Private institution	**2.25**	[1.04–4.87]	0.04
V. Prefer ICTs to traditional channels of communication	Age > 33 years	**0.40**	[0.215–0.725]	<.001
	Specialists	0.94	[0.522–1.69]	0.82
VI. ICTs are useful for promoting my professional services	Age > 33 years	**0.01**	[0–0.139]	<.001
	Years of Experience > 8	2.36	[0.313–13.2]	0.35
	Specialists	**5.37**	[1.81–20]	0.01
	Private institution	**5.48**	[2.05–16.9]	<.001
VII. ICTs can be useful for resolving clinical cases	Age > 33 years	0.53	[0.256–1.05]	0.07
	Private institution	**2.51**	[1.28–4.96]	0.01

Notes: Reference age category is < 33 years old, years of experience < 8 years, gender category is male, type of practice is general dentist, and type of institution is public. *ICT* information and communication technology, *CI* confidence interval

Age was also identified as an important factor, since dentists older than 33 years of age were more likely to prefer traditional channels of communication rather than ICTs, and presented higher odds of reporting privacy and security concerns (OR 1.84), lack of mobile internet access (OR 2.14), and lack of time to learn and/or use ICTs (OR 1.94) (Table 3). Further analysis between demographic variables and their association with the perceptions and barriers studies are depicted on Supplemental Appendix Tables S2 and S3.

**Table 3:** Generalized logistic regression analysis between specific barriers of ICTs and demographic variables among Ecuadorian dentists

Barrier	Variable	Odds Ratio	95% CI	*p*-value
I. Privacy or security concerns about personal and/or patient information	Age > 33 years	**1.84**	[1.16–2.94]	0.01
	Female gender	**1.89**	[1.19–3.03]	0.01
II. Lack of access to mobile internet	Age > 33 years	**2.14**	[1.22–3.8]	0.01
	Specialists	**0.35**	[0.182–0.635]	<.001
	Female gender	**1.91**	[1.13–3.25]	0.02
	Private institution	0.79	[0.451–1.41]	0.42
III. Lack of internet access at work	Specialists	**0.37**	[0.206–0.653]	<.001
	Female gender	**2.1**	[1.28–3.49]	<.001
	Private institution	**0.6**	[0.356–1]	0.05
IV. Lack of time to neither learn how to use or use ICTs	Age > 33 years	**1.94**	[1.24–3.05]	<.001
	Specialists	**0.5**	[0.316–0.797]	<.001

Notes: Reference age category is < 33 years old, gender category is male, type of practice is general dentist, and type of institution is public. *ICT* information and communication technology, *CI* confidence interval

## Discussion

The purpose of this study was to assess the use, perceptions, and barriers of ICTs among a sample of Ecuadorian dentists. Overall, we found that virtually all surveyed participants used at least one ICT platform on their daily practice. This shows that dentistry has adopted the use of ICTs to share deals and promotions, networking, providing customer service, and advertising dental practice with patients. Previous studies assessing the use of social media among dentists, have found that between 51 to 76% of individuals used ICTs for professional purposes [16]. In our study, 95.3% of respondents stated they used ICTs for this end. On the other hand, previous publications have reported that patients tend to give higher satisfaction scores when dental care providers use ICTs for patient communication [17]. In this regard, our study found that most dentists (96.2%) already used ICTs to communicate with patients, with WhatsApp cited as the most used application for this purpose (83.9%). The latter finding agrees with a previous study in which roughly 70% of surveyed health professionals perceived this application as beneficial during practice [18].

Currently, Facebook is considered as the biggest social media platform, allowing for complex social interactions between individuals with similar interests [19]. In agreement with recent studies, we found that this platform was the most used ICT for interacting with both patients (67.8%), and colleagues (88%) [19]. The widespread adoption of this platform makes it an attractive mean of interacting with patients and efficiently sharing dental care information. However, Facebook has been widely criticized recently for mismanaging user information, which should be of concern considering that more than two thirds of those surveyed in our study had privacy or security concerns about personal and/or patient information related to the use of ICTs for the dental practice [20].

The internet has become an essential tool for healthcare professionals to improve their knowledge and to acquire updated information about health care and their profession. In our study, roughly two-thirds of respondents used ICTs to search for academic information. Still, there are important questions regarding the quality of online information that ends up being transformed into practice. Systematic reviews and metanalysis provide the strongest evidence for assessing original works, however only 7% of the participants in our study were found to frequently use them, a finding that relates to a previous study among physicians [21]. On the other hand, most dentists surveyed considered that ICTs can be useful for health promotion, and for self-promoting professional services. ICT platforms allow for an ever-increasing amount of data to be shared, transforming the way patients approach and manage their care. This in turn leads to changes in patient’s expectations, which dentists must be able to identify and adapt in order to be able to leverage them into an increased patient satisfaction [22].

On the subject of barriers, privacy and patient confidentiality have been regarded as the most significant difficulties to m-Health implementation in regions such as Europe and America [23]. Similarly, we found that privacy and security concerns related to personal and or patient information were the biggest concerns related to ICTs in more than two-thirds of the Ecuadorian dentists surveyed. The issue of patient privacy expands well beyond the domain of the dentistry practice, this is why it is essential for both ICT providers and healthcare practitioners to work together in order to ensure that patient information is only accessed by authorised individuals [22]. Furthermore, possible solutions for securing many aspects of patient-related information are currently being explored by researchers to ensure privacy and confidentiality [24].

In a previous study conducted in 2013 among Latin American dentists, around 80% of participants reported lacking a reliable internet connection [25]. In contrast, we found that less than a third of Ecuadorian dentists considered the lack of a reliable mobile connection and internet at work as barriers for ICT implementation. A possible explanation might be attributed to the ongoing expansion of wireless technology infrastructure across Latin American countries [6]. Still, there is a considerable proportion of dentists that could miss potential interventions through ICTs due to a lack of widespread access to the internet. In developing societies, the adoption of new technologies often yields increased benefits by leapfrogging the need to implement infrastructure and enabling the distributed implementation of new strategies [26, 27].

Finally, we found several associations between demographic variables and the perception towards ICTs. For instance, dentists working at private practices were more likely to agree on the usefulness of ICTs to promote their professional services, collaborate with other colleagues, and for solving clinical cases than their counterpart. This can be related to the need of private dentists to adapt to evolving patient preferences, and the potential of ICTs to offer tools which provide a competitive advantage to peers [28]. In contrast, older dentists had a less favourable view to most of the perceptions studied and were more likely to report barriers related to the use of ICTs. A possible explanation can be attributed to what is known as the digital divide [29]. This concept relates to the observation that users born in the digital era are more familiar with the internet and computers in general, therefore they tend to adopt technologies at a faster rate than their older peers [30].

In conclusion, our study found a high use of ICTs among Ecuadorian dentists, a finding that compares to previous reports among physicians and nurses in the country [13, 31]. But despite the potential benefits of these technologies, there are many barriers and factors that need to be accounted when implementing ICTs. In particular, it is necessary to improve and increase the use of evidence-based resources among dentists, and the implementation of these resources in postgraduate and continuing dental education.

### Strengths and limitations

One of the main strengths of this study was that it covered a relatively large sample size of dentists, with participants of different age, gender, and practice. However, in light of our findings there are several limitations worth mentioning. First, this study was not conducted in all Latin American countries, as such the preferences and usage of ICTs could differ, limiting the ability to generalize our results to all Spanish-speaking dentists. Second, due to the cross-sectional nature of the study, cause and effect relationships cannot be made. Third, the original validation of the survey used in this study was conducted among physicians, it should be noted that validity and reliability coefficients have not been calculated yet specifically for dentists. Fourth, although sources of bias were minimized wherever possible in the study design, the frequencies, perceptions, and barriers may be influenced by confounding variables not accounted for in this study. Also, participants knew the purpose of the study, which could have affected the answers some of them gave. Finally, although the use of median splits for variables is an actively debated topic among statisticians, we used this method after a thorough consideration of the statistical power based on sample size, minimizing the possibility of incurring in a type II error [32–34]. To the best of our knowledge, this is the first study to assess the use, preferences, and perceptions related to ICTs among Ecuadorian dentists.

## Conclusions

The use of information and communication technologies provide the tools for enabling fast and reliable real-time communications, as well as the transfer of information between dental professionals and their patients. In our study, we found that Ecuadorian dentists had a high usage rate of ICTs, mainly for communicating with other colleagues and patients, while the academic use of technology remains a comparatively underused application. Most of the participants surveyed had a positive perception towards ICTs, while privacy and security concerns about personal and/or patient information remains the main barrier for ICT adoption according to our survey. Older age was associated with a less favourable perception toward ICTs, as well as an increased likelihood of reporting barriers to the use of technology. Further studies are needed to expand on our findings and the issues identified, in order to accelerate the adequate adoption of ICTs in the dental care practice for the benefit of the patients.

## Supplementary information

**Additional file 1: Supplemental Appendix Table S1.** Frequency of use of specific ICTs classified by intended use **Table S2:** Logistic regression analysis between demographic variables and specific perceptions related to ICTs among Ecuadorian dentists. **Table S3:** Logistic regression analysis between demographic variables and specific barriers related to ICTs among Ecuadorian dentists.

## Data Availability

The datasets used and/or analyzed during the current study are available from the corresponding author on reasonable request.

## Abbreviations

ICTs: Information and communication technologies
SMS: Short message service
CEISH: Comité de ética e Investigación en Seres Humanos
