# Supplementary material for: The use of information and communication technologies in Latin American dentists: a cross-sectional study from Ecuador
Source: BMC Oral Health. 2020 May 19;20:146. doi: 10.1186/s12903-020-01137-z (PMC7236194; doi:10.1186/s12903-020-01137-z)
Supplement: Supplementary file 1 — Additional file 1: Supplemental Appendix Table S1. Frequency of use of specific ICTs classified by intended use Table S2: Logistic regression analysis between demographic variables and specific perceptions related to ICTs among Ecuadorian dentists. Table S3: Logistic regression analysis between demographic variables and specific barriers related to ICTs among Ecuadorian dentists. [file 12903_2020_1137_MOESM1_ESM.docx]

**Supplemental Appendix**

**Table S1.** Frequency of use of specific ICTs classified by intended use

|  | **Frequency** n (%) |  | **Frequency** n (%) |
| --- | --- | --- | --- |
| **Communicating with colleagues** | | **Communicating with Patients** | |
| WhatsApp | 333 (97.4) | WhatsApp | 320 (93.6) |
| Mail | 320 (93.6) | Mail | 240 (70.2) |
| SMS | 196 (57.3) | SMS | 235 (68.7) |
| Facebook Messenger | 195 (57.0) | Facebook Messenger | 185 (54.1) |
| Line | 23 (6.7) | Line | 10 (2.9) |
| Hangouts | 21 (6.1) | Telegram | 4 (1.2) |
| Telegram | 7 (2.0) | Hangouts | 4 (1.2) |
| Vibe | 6 (1.8) | Vibe | 3 (0.9) |
| **Interacting with colleagues** | | **Interacting with Patients** | |
| Facebook | 301 (88.0) | Facebook | 232 (67.8) |
| YouTube | 267 (78.1) | Instagram | 198 (57.9) |
| Instagram | 261 (76.3) | YouTube | 73 (21.3) |
| Twitter | 139 (40.6) | Twitter | 61 (17.8) |
| Google Photos | 63 (18.4) | Google Photos | 26 (7.6) |
| Snapchat | 38 (11.1) | Snapchat | 18 (5.3) |
| Blogger | 11 (3.2) | Blogger | 3 (0.9) |
| Tumbler | 8 (2.3) | Tumbler | 0 (0.0) |
| **Academic purposes** | |  |  |
| PubMed | 146 (42.7) |  |  |
| Medscape | 87 (25.4) |  |  |
| Google Scholar | 80 (23.4) |  |  |
| Academia.edu | 75 (21.9) |  |  |
| Cochrane | 49 (14.3) |  |  |
| Scopus | 43 (12.6) |  |  |
| ResearchGate | 40 (11.7) |  |  |
| UpToDate | 15 (4.4) |  |  |

**Table S2:** Logistic regression analysis between demographic variables and specific perceptions related to ICTs among Ecuadorian dentists.

| **Variable** | | **ICTs useful to search for new work opportunities** | | | | | **ICTs useful for continuing dental education** | | | | | | | | |
| --- | --- | --- | --- | --- | --- | --- | --- | --- | --- | --- | --- | --- | --- | --- | --- |
|  |  | OR | | | CI | *p*-value | OR | | | | CI | | *p*-value | | |
| Age > 33 years | | **0.209** | | | **(0.046-0.96)** | **0.0434*** | **0.091** | | | | **(0.019-0.462)** | | **0.00303**** | | |
| Years of practice > 8 | | 1.79 | | | (0.405-7.21) | 0.4267 | **4.78** | | | | **(0.978-22.1)** | | **0.0452*** | | |
| Specialist Dentist | | 1.33 | | | (0.56-3.28) | 0.5279 | 2.85 | | | | (1.06-8.88) | | 0.05014. | | |
| Rural Area | | NA | | | NA | NA | 2.48 | | | | (0.406-48.4) | | 0.41282 | | |
| Female gender | | 0.429 | | | (0.17-0.99) | 0.0569 | 0.562 | | | | (0.225-1.32) | | 0.1967 | | |
| Private Practice | | 1.5 | | | (0.605-3.63) | 0.3741 | 2.17 | | | | (0.852-5.66) | | 0.10695 | | |
| **Variable** | **ICTs useful for health promotion** | | | | | | | **ICTs useful for working in group** | | | | | | |  |
|  | OR | | | | CI | *p*-value | | OR | | | CI | | *p-*value | |  |
| Age > 33 years | 0.424 | | | | (0.102-1.70) | 0.23244 | | 0.546 | | | (0.123-2.55) | | 0.43773 | |  |
| Years of practice > 8 | 0.866 | | | | (0.216-3.30) | 0.83627 | | 1.37 | | | (0.292-6.1) | | 0.68485 | |  |
| Specialist Dentist | 1.45 | | | | (0.647-3.38) | 0.3734 | | 1.97 | | | (0.821-5.15) | | 0.14382 | |  |
| Rural Area | NA | | | | NA | NA | | 1.28 | | | (0.297-9.09) | | 0.76396 | |  |
| Female | **0.167** | | | | **(0.055-0.42)** | **0.00041**** | | **0.288** | | | **(0.11-0.673)** | | **0.00634**** | |  |
| Private Practice | 1.25 | | | | (0.525-2.89) | 0.60552 | | 1.85 | | | (0.776-4.4) | | 0.16139 | |  |
| **Variable** | **Prefer ICTs to Traditional Channels** | | | | | | | | **ICTs useful for solving cases** | | | | | |  |
|  | OR | | | CI | | *p*-value | | | OR | | | CI | | *p*-value |  |
| Age > 33 years | **0.162** | | | **(0.049-0.496)** | | **0.00187**** | | | **0.206** | | | **(0.053-0.819)** | | **0.023*** |  |
| Years of practice > 8 | **3.32** | | | **(1.1-10.7)** | | **0.03706*** | | | 3.12 | | | (0.784-12.1) | | 0.1011 |  |
| Specialist Dentist | 0.809 | | | (0.429-1.54) | | 0.51464 | | | **2.3** | | | **(1.04-5.46)** | | **0.0469*** |  |
| Rural Area | 1.35 | | | (0.402-6.25) | | 0.6551 | | | 1.33 | | | (0.374-6.41) | | 0.6829 |  |
| Female | 0.781 | | | (0.425-1.42) | | 0.42037 | | | 0.502 | | | (0.24-1.01) | | 0.0587 |  |
| Private Practice | 1.67 | | | (0.862-3.21) | | 0.12559 | | | **2.35** | | | **(1.1-5.06)** | | **0.027*** |  |
| **Variable** | | | **Service Self Promotion** | | | | | | |  |  |  |  |  |  |
|  |  |  | OR | | CI | *p*-value | | | |  |  |  |  |  |  |
| Age > 33 years | | | **0.012** | | **(0-0.15)** | **0.00119**** | | | |  |  |  |  |  |  |
| Years of practice > 8 | | | 3.52 | | (0.421-23.5) | 0.20487 | | | |  |  |  |  |  |  |
| Specialist Dentist | | | **5.03** | | **(1.66-19.1)** | **0.00793**** | | | |  |  |  |  |  |  |
| Rural Area | | | NA | | NA | NA | | | |  |  |  |  |  |  |
| Female | | | 1.1 | | (0.462-2.68) | 0.82477 | | | |  |  |  |  |  |  |
| Private Practice | | | **5.84** | | **(2.11-19.1)** | **0.00139**** | | | |  |  |  |  |  |  |

*Notes: NA denotes insufficient instances for regression analysis. Significance codes: *p < .05. **p < .01. ***p < .001*

**Table S3:** Logistic regression analysis between demographic variables and specific barriers related to ICTs among Ecuadorian dentists.

| **Variable** | **Privacy and security concerns** | | | | **Lack of time to learn or use ICTs** | | |
| --- | --- | --- | --- | --- | --- | --- | --- |
|  | OR | | CI | *p*-value | OR | CI | *p*-value |
| Age > 33 years | **4.52** | | **(1.89-11.8)** | **0.00109**** | 1.88 | (0.831-4.38) | 0.13186 |
| Years of practice > 8 | **0.405** | | **(0.156-0.962)** | **0.04855*** | 0.942 | (0.406-2.14) | 0.8874 |
| Specialist Dentist | 0.702 | | (0.422-1.16) | 0.17111 | **0.524** | **(0.32-0.849)** | **0.00922**** |
| Rural Area | 1.06 | | (0.41-2.93) | 0.90487 | **0.303** | **(0.102-0.8)** | **0.02139*** |
| Female gender | **0.52** | | **(0.322-0.83)** | **0.00662**** | 0.73 | (0.465-1.14) | 0.16923 |
| Private Practice | 1.22 | | (0.701-2.1) | 0.48679 | 0.774 | (0.459-1.3) | 0.33545 |
| **Variable** | | **Difficult Internet Access** | | | **No Internet Access** | | |
|  |  | OR | CI | *p*-value | OR | CI | *p*-value |
| Age > 33 years | | 1.48 | (0.523-4.31) | 0.462817 | 2 | (0.688-6.02) | 0.2103 |
| Years of practice > 8 | | 1.3 | (0.448-3.74) | 0.62343 | 1.17 | (0.39-3.47) | 0.77341 |
| Specialist Dentist | | **0.309** | **(0.165-0.558)** | **0.000151***** | **0.347** | **(0.182-0.638)** | **0.00091***** |
| Rural Area | | 0.873 | (0.3-2.34) | 0.793732 | 1.25 | (0.431-3.36) | 0.66625 |
| Female gender | | **0.45** | **(0.267-0.748)** | **0.002307**** | **0.528** | **(0.309-0.891)** | **0.01779*** |
| Private Practice | | 0.738 | (0.415-1.32) | 0.301758 | 0.834 | (0.463-1.52) | 0.54909 |

*Notes: Significance codes *p < .05. **p < .01. ***p < .001*
